# Understanding Antimicrobial Self-Medication Practices in the Andaman and Nicobar Islands in India

**DOI:** 10.7759/cureus.80473

**Published:** 2025-03-12

**Authors:** Yashwant Sridhar, Aanchal Anand, Ajay Sethuraman, Samar Hossain

**Affiliations:** 1 Community Medicine, Andaman & Nicobar Islands Institute of Medical Sciences, Sri Vijaya Puram, IND

**Keywords:** andaman and nicobar islands, antimicrobial resistance, drug misuse, rural community, self-medication behaviors

## Abstract

Background

Antimicrobial resistance (AMR) is a growing global health concern, primarily driven by the irrational and excessive use of antimicrobials. Self-medication with antibiotics, often without a medical prescription, contributes significantly to the emergence of resistant bacteria strains. This study aims to assess the patterns of antimicrobial consumption and its role in the development of antibiotic resistance.

Aim and objective

The aim and objective of the study are to evaluate the prevalence, determinants, and associated factors of self-medication with antimicrobials in a rural area of the Andaman and Nicobar Islands.

Materials and methods

A cross-sectional study was conducted among 437 participants in the community. Data on socio-demographic characteristics, self-medication patterns, and reasons for antimicrobial use were collected using a structured questionnaire. The data was analyzed by using SPSS version 26.0 (IBM Corp., Armonk, NY, US) to assess associations between self-medication and socio-demographic variables.

Results

The study found that 64.3% of participants self-medicated with antimicrobials, while 35.7% did not. The most common sources for determining the dosage included pharmacists (27.8%), hospital staff (21.7%), and personal experience (19.3%). A significant proportion (65.4%) altered the dosage during the course of treatment, primarily due to symptom improvement (53.4%) or lack of improvement (25.1%). A statistically significant association was observed between self-medication and age (p = 0.024), gender (p = 0.0095), religion (p = 0.00), and socio-economic status (p = 0.01).

Conclusion

High rates of self-medication with antimicrobials, coupled with inappropriate usage patterns, contribute to the increasing burden of antibiotic resistance. Strengthening public awareness, implementing stricter regulations on antimicrobial sales, and promoting rational prescribing practices are essential measures to combat AMR.

## Introduction

Antimicrobial resistance (AMR) has emerged as a critical global health threat, undermining the efficacy of treatments for various infectious diseases. The World Health Organization (WHO) identifies AMR as one of the top public health challenges worldwide, attributing its rise to the misuse and overuse of antimicrobials in humans, animals, and agriculture [1].

In developing countries like India, the rising burden of AMR, driven by the overuse and misuse of antibiotics, presents a particularly alarming public health crisis. Factors such as the over-the-counter availability of antibiotics, lack of stringent regulatory frameworks, and limited public awareness contribute to widespread self-medication practices [2]. A community-based cross-sectional study from central India reported a high prevalence of self-medication, highlighting it as a significant factor in the development of AMR [3].

The ease of access to antibiotics without prescriptions in India exacerbates the problem. Many pharmacies dispense antibiotics without proper medical guidance, leading to inappropriate usage [4]. This unregulated consumption fosters the emergence of resistant bacterial strains, complicating treatment protocols and increasing healthcare costs.

Globally, self-medication with antibiotics (SMAs) is a widespread concern, with prevalence varying significantly across regions. A systematic review reported rates ranging from 12.8% to 77.1%, depending on factors such as healthcare access and regulatory frameworks [5]. Such practices are often driven by the desire for quick relief, previous successful experiences, and the perceived high cost of medical consultations.

The consequences of irrational antimicrobial use are profound. They include treatment failures, prolonged illness, increased mortality, and the spread of resistant infections [6]. Addressing this issue requires a multifaceted approach, encompassing public education, stricter enforcement of prescription regulations, and improved access to healthcare services.

This study aims to evaluate the prevalence, determinants, and associated factors of self-medication with antimicrobials in a rural Indian community. By understanding these patterns, we can develop targeted interventions to mitigate the contribution of self-medication to antibiotic resistance.

## Materials and methods

Study design

This is a community-based cross-sectional study.

Study area

The study was conducted in a rural community setting in the Chouldari area of Sri Vijaya Puram, the capital of the Andaman and Nicobar Islands in India.

Study population

The study population consists of all adults residing in Chouldari village of Sri Vijaya Puram in South Andaman district, Andaman and Nicobar Islands, India.

Inclusion Criteria

The inclusion criteria include adults aged 18 years and older who have been residing in the community for a minimum of one year and who provided consent to be a part of the study.

Exclusion Criteria

The exclusion criteria include individuals with severe cognitive impairment to comprehend the questionnaire or who are healthcare workers (HCWs) themselves or have HCWs as family members residing with them.

Sample size

For the calculation of the sample size, we used the formula:

 $N= Z^{2}(1-&alpha;/2) *P(1-P)/d^{2}$

where N = sample size, Z (1 - α/2) = 1.96 value of the standard normal, variate corresponds to the level of significance α of 0.5, d = specified precision on either side of the mean, and P = prevalence rate of self-medication.

As no prior study had been conducted in the Andaman and Nicobar Islands, the prevalence was assumed to be P = 50%. With d = 5%, the calculated sample size was N = 384. Considering a 10% non-response rate, a minimum of 430 subjects were required for inclusion in the study.

Sampling method

The sampling method was systematic random sampling for household selection and simple random sampling for the selection of participants.

Methodology

The list of households was obtained from the population register maintained at the Rural Health Training Centre (RHTC), Chouldari, Sri Vijaya Puram. To identify the study population, all households listed in the records were screened based on the predefined inclusion criteria. Once eligible households were identified, systematic random sampling was employed to ensure an unbiased selection process. A sampling interval was determined by dividing the total number of eligible households by the required sample size. The first household in the sequence was selected through simple random sampling using a computer-generated random number.

From each selected household, only one eligible participant was chosen at random using the same random number method to ensure equal representation within the sample. If a household was found locked during the initial visit, two additional follow-up visits were made at different times of the day to maximize the chances of contact. If the household remained inaccessible after three consecutive visits, it was excluded from the study, and the next eligible household in the sequence was selected.

All interviews were conducted in the participants' homes, ensuring their privacy by requesting a separate room for the duration of the interview. Face-to-face interactions with the investigator facilitated data collection on self-medication practices with antimicrobials. The prevalidated pilot-tested questionnaire was administered directly by the study investigators, who are trained healthcare professionals specializing in public health. Informed consent was obtained prior to each interview, ensuring ethical compliance.

The data collection process lasted approximately 20-25 minutes to respect participants' time while capturing comprehensive information. The structured questionnaire was divided into two key sections. The first section gathered socio-demographic details, including participant age, gender, marital status, religion, education, and income. The second section focused on patterns and reasons for self-medication with antimicrobials.

Ethical considerations

The Institutional Ethics Committee, Andaman & Nicobar Islands Institute of Medical Sciences (ANIIMS), Sri Vijaya Puram, India, issued approval, ANIIMS/IRSC/2021-22/88. Written informed consent was obtained from all study participants after providing a detailed explanation of the study's nature and purpose.

Statistical analysis

Data were entered into Microsoft Excel (Microsoft Corp., Redmond, WA, US) and analyzed using the Statistical Package for Social Sciences (SPSS), PC version 26 (IBM Corp., Armonk, NY, US). Categorical variables were presented as frequencies and percentages. The chi-squared test was applied to assess differences in proportions, with a two-tailed p-value < 0.05 considered statistically significant.

## Results

The socio-demographic characteristics of the study participants are summarized in Table 1. This study included a total of 437 participants. The majority (n = 283, 64.7%) belonged to the 31-59-year age group, followed by 18-30 years (n = 108, 24.7%) and ≥60 years (n = 46, 10.5%). A higher proportion of participants were men (n = 246, 56.2%), while women accounted for 43.5% (n = 190), and one participant (n = 1, 0.2%) identified as transgender. Regarding marital status, three-fourths of the participants were married (n = 332, 75.9%), 22.2% (n = 97) were unmarried, and 1.8% (n = 8) were divorced, separated, or widowed. The majority followed Hinduism (n = 366, 83.7%), while Muslims (n = 45, 10.3%) and Christians (n = 26, 5.9%) comprised the remaining participants. Educational qualifications varied, with 21.7% (n = 95) having completed 12th standard, followed by 19.7% (n = 86) with middle school education and 15.1% (n = 66) with 10th standard education. A small proportion of the study population was illiterate (n = 26, 5.9%), while postgraduate qualifications were held by only 1.6% (n = 6). Socio-economic classification based on the Modified Kuppuswamy Scale showed that the majority belonged to the upper class (n = 329, 75.3%), followed by the upper middle class (n = 50, 11.4%), middle class (n = 25, 5.7%), lower middle class (n = 17, 3.8%), and lower class (n = 16, 3.8%).

**Table 1:** Socio-Demographic Characteristics of the Study Participants

Socio-demographic variable	Categories	Frequency	Percentage
Age in years	18-30	108	24.8
	31-59	283	64.7
	≥60	46	10.5
Gender	Female	190	43.6
	Male	246	56.2
	Transgender	1	0.2
Marital status	Married	332	75.9
	Divorced/separated/widowed	8	1.8
	Unmarried	97	22.3
Religion	Christian	26	5.9
	Hindu	366	83.7
	Muslim	45	10.4
Educational qualification	Illiterate	26	5.9
	Primary school	46	10.5
	Middle school	86	19.6
	10th pass	66	15.1
	12th pass	95	21.7
	Higher secondary	49	11.2
	Graduate	63	14.4
	Postgraduate	6	1.6
Socio-economic status (Modified Kuppuswamy Scale)	Upper class	329	75.2
	Upper middle class	50	11.4
	Middle class	25	5.7
	Lower middle class	17	3.8
	Lower class	16	3.9

The patterns and reasons for self-medication with antimicrobials are detailed in Table 2. Among the 437 study participants, 64.3% (n = 281) reported self-medicating with antimicrobials, while 35.7% (n = 156) did not. The most common basis for dosage determination was consulting a pharmacist (n = 78, 27.8%), followed by hospital staff or nurses (n = 61, 21.7%), and personal experience (n = 54, 19.3%). Other sources included internet searches (n = 43, 15.3%), recommendations from family or peers (n = 25, 8.8%), and referring to prescribing information (n = 20, 7.1%). In terms of dosage modifications, 65.4% (n = 184) altered the dosage occasionally, while 22.6% (n = 63) frequently changed it, and only 12% (n = 34) strictly adhered to the initial dosage. The most cited reason for dosage change was symptom improvement (n = 132, 53.4%), followed by lack of symptomatic relief or worsening of illness (n = 62, 25.1%), and experiencing adverse effects (n = 53, 21.5%). Regarding antimicrobial switching, 59.4% (n = 167) never switched antimicrobials, while 30.2% (n = 85) did so occasionally, and 10.4% (n = 29) always substituted one antimicrobial for another. The primary reasons for switching antimicrobials were perceived lack of efficacy of the initial drug (n = 25, 21.9%), exhaustion of the initial medication (n = 29, 25.4%), and cost-related considerations (n = 41, 35.9%). Regarding treatment discontinuation, 19.2% (n = 54) completed the full antimicrobial course, while 27.5% (n = 77) stopped medication upon symptom resolution. A significant proportion (n = 64, 22.7%) discontinued the medication within a few days, regardless of the outcome, whereas 30.6% (n = 86) stopped when they ran out of medication. The most common conditions leading to self-medication included respiratory tract infections (n = 136, 20%), fever (n = 118, 17.3%), skin infections (n = 76, 11.3%), and gastrointestinal infections (n = 48, 7%).

**Table 2:** Patterns and Reasons for Self-Medication With Antimicrobials GIT: gastrointestinal tract

Have you ever self-medicated with antimicrobials?		
Yes	281	64.3
No	156	35.7
How do you decide the dosage of antimicrobials to be taken?		
By checking the prescribing information	20	7.1
Consulting any known nurse/hospital staff	61	21.7
Consulting peers-family/friends	25	8.8
Consulting pharmacist	78	27.8
Internet	43	15.3
Previous experience	54	19.3
Did you ever change the dosage of antimicrobials during the course of self-medication?		
Never	34	12.0
A few times	184	65.4
Always	63	22.6
Why did you change the dosage of antimicrobials during the course of self-medication?		
Disease did not improve/worsened	62	25.1
Symptoms improved	132	53.4
Side effects	53	21.5
Have you ever changed the antimicrobial you were taking during the course of self-medication?		
Never	167	59.4
Sometimes	85	30.2
Always	29	10.4
Why did you change the antimicrobial drug during self-medication?		
Pharmacy ran out of former antimicrobial	14	12.2
The former antimicrobial did not work	25	21.9
The former antimicrobial was over	29	25.4
The latter one was cheaper	41	35.9
Others	5	4.6
When did you stop taking antimicrobials?		
After the antimicrobial was over	86	30.6
After completing the course of antimicrobial	54	19.2
After a few days regardless of the outcome	64	22.7
After symptoms disappeared	77	27.5
For which of the following diseases did you self-medicate with antimicrobials? Categories are not mutually exclusive of each other		
Fever	118	17.3
Respiratory tract infections	136	20.0
Cough	59	8.7
Sore throat	34	5.0
Runny nose	43	6.4
GIT	48	7.0
Diarrhea	34	5.0
Vomiting	14	2.0
Ear pain	21	3.0
Eye infection	39	5.8
Genito-urinary infections	46	6.8
Trauma wound	11	1.7
Skin diseases	76	11.3

The associations between socio-demographic variables and self-medication behaviors are shown in Table 3. A statistically significant association was observed between age and self-medication (χ² = 16.65, p = 0.024). The 18-30-year age group had a higher prevalence of self-medication (66 cases) compared to older adults (≥60 years), where non-self-medication was more common (28 cases). Gender also exhibited a statistically significant relationship with self-medication (χ² = 18.50, p = 0.0095). Men (179 cases) were more likely to self-medicate compared to women (101 cases). The single transgender participant also reported self-medicating. No significant association was observed between marital status and self-medication (χ² = 2.62, p = 0.29), although a greater proportion of married individuals (219 cases) reported self-medication compared to unmarried participants. Religion demonstrated a highly significant association with self-medication behavior (χ² = 16.93, p = <0.001), with Hindus exhibiting the highest self-medication rates (243 cases), followed by Muslims (31 cases) and Christians (seven cases). Although educational qualification did not show a statistically significant relationship with self-medication (χ² = 34.62, p = 1.31), participants with higher secondary education (14 cases) and graduates (43 cases) engaged in self-medication at varying levels. Socio-economic status was significantly associated with self-medication (χ² = 12.77, p = 0.01), with higher self-medication rates among the upper class (199 cases) compared to the lower class (nine cases).

**Table 3:** Association Between Socio-Demographic Variables and Self-Medication With Antimicrobials p < 0.05 is considered statistically significant

Socio-demographic variable	Categories	Yes (n = 281) (%)	No (n = 156) (%)	Chi-squared value	p-value
Age in years	18-30	66 (23.4)	42 (26.9)	16.65	0.024
	31-59	197 (70.1)	86 (55.2)		
	≥60	18 (6.5)	28 (17.9)		
Gender	Female	101 (35.9)	89 (57.1)	18.50	9.5
	Male	179 (63.7)	67 (42.9)		
	Transgender	1 (0.4)	0 (0.0)		
Marital status	Married	219 (77.9)	113 (72.5)	2.62	0.29
	Divorced/separated/widowed	6 (2.2)	2 (12.9)		
	Unmarried	56 (19.9)	41 (14.6)		
Religion	Christian	7 (2.5)	19 (12.3)	16.93	0.00
	Hindu	243 (86.5)	123 (78.8)		
	Muslim	31 (11.0)	14 (8.9)		
Educational qualification	Illiterate	16 (5.6)	10 (6.5)	34.62	1.31
	Primary school	29 (10.3)	17 (10.9)		
	Middle school	63 (22.4)	23 (14.8)		
	10th pass	50 (17.8)	16 (10.3)		
	12th pass	62 (22.1)	33 (21.1)		
	Higher secondary	14 (4.9)	35 (22.4)		
	Graduate	43 (15.4)	20 (12.8)		
	Postgraduate	4 (1.5)	2 (1.2)		
Socio-economic status (Modified Kuppuswamy Scale)	Upper class	199 (70.9)	130 (83.3)	12.77	0.01
	Upper middle class	42 (14.9)	8 (5.2)		
	Middle class	19 (6.8)	6 (3.8)		
	Lower middle class	12 (4.2)	5 (3.2)		
	Lower class	9 (3.2)	7 (4.5)		

## Discussion

The present study found that 64.3% of participants self-medicated with antimicrobials, with the most common sources for dosage determination being pharmacists (27.8%) and hospital staff (21.7%). In a cross-sectional descriptive study conducted by Donkor et al. in Ghana among tertiary students, 70% of participants reported SMAs [7]. Likewise, a cross-sectional study by Gebregziabher et al. in Eritrea found that 67.1% of college students engaged in SMAs [8]. However, our study population, consisting of a broader community sample, exhibited even higher rates compared to studies focusing on educated groups. This suggests that antibiotic self-medication is not limited to educated individuals but is widespread across all socio-demographic segments in rural India.

Our study also observed that 65.4% of participants altered antibiotic dosages, with symptom improvement (53.4%) being the most common reason for modification. In a systematic review and meta-analysis conducted by Ocan et al. in Nigeria in 2015, over 60% of respondents discontinued antibiotics once symptoms improved [9]. However, in high-income countries like Sweden, a cross-national study by Cars et al. in 2001 reported that only 30% of individuals discontinued antibiotics due to symptom relief [10]. The discrepancy suggests that awareness campaigns and stricter antimicrobial stewardship programs are crucial in low-resource settings like India.

More than one-third (35.9%) of participants enrolled in the study switched antibiotics due to cost considerations, indicating economic constraints as a major determinant of antimicrobial use patterns. In a cross-sectional study by Llor and Bjerrum in 2014, economic constraints played a much smaller role in determining antimicrobial choices in insured populations [11]. The high cost-driven antibiotic switching in rural India underscores the need for subsidized antibiotic access and price control policies to ensure appropriate usage.

Another key finding was that the majority of respondents in our study (27.8%) relied on pharmacists for antibiotic recommendations. However, in a cross-national database study by Goossens et al. in 2005, pharmacist-driven antibiotic recommendations in Germany were below 5%, reflecting stricter legal frameworks that regulate over-the-counter antibiotic dispensing [12]. These differences highlight the urgent need for policy interventions to regulate pharmacist antibiotic dispensing practices in India.

The findings of our study are further supported by a systematic review and meta-analysis by Torres et al. in 2018, which identified factors influencing SMAs in low- and middle-income countries, including ease of access, affordability, and lack of regulation [13]. These factors are also evident in our study population, where antibiotics were often obtained without prescriptions (n = 61, 21.7%) and used based on previous experience or peer recommendations (n = 54, 19.3%).

Additionally, a study by Sezerano and Niyonkuru in 2024 highlights that self-medication is a key driver of antibiotic resistance, with a pooled global prevalence of 27.7% [14]. The significantly higher prevalence in our study (64.3%, n = 281) reinforces the urgent need for public awareness campaigns, stricter regulations, and improved healthcare accessibility to combat irrational antimicrobial use and its consequences.

Furthermore, a study by Ganguly et al. in 2020 focusing on pediatric populations in tertiary care hospitals found similar patterns of SMAs, emphasizing the risk of inappropriate antibiotic use in vulnerable groups [15]. This aligns with our findings that demonstrate self-medication as a widespread practice across different age groups.

Our study's high prevalence of antimicrobial self-medication, coupled with patterns of dosage modification, discontinuation, and switching antibiotics, underscores the importance of implementing community-level interventions to improve antibiotic use behaviors. Future research should focus on intervention-based studies to assess the efficacy of public health measures in reducing self-medication and its contribution to AMR.

Strengths

This study provides valuable insights into the prevalence and determinants of SMAs in a rural Indian population, contributing to the limited body of research in the Andaman and Nicobar Islands. The substantial sample size of 437 participants enhances the generalizability of findings within the studied population. However, certain limitations must be acknowledged.

Limitations

The cross-sectional study design restricts the ability to infer causal relationships between SMAs and their contributing factors. Additionally, self-reported data may be influenced by recall bias and social desirability bias, potentially leading to underreporting or overreporting of SMA behaviors. The study was conducted in a specific rural setting, limiting its applicability to urban populations or other regions with varying healthcare access and regulatory frameworks. Lastly, the absence of microbiological testing prevents direct assessment of AMR patterns arising from SMA practices.

## Conclusions

This study underscores the multifaceted issue of self-medication with antimicrobials and its significant implications for AMR. The high prevalence observed coupled with associations with socio-demographic variables emphasizes the urgent need for targeted interventions. Strengthening regulatory policies, improving healthcare accessibility, and fostering community-driven educational programs are pivotal steps toward mitigating the risks associated with SMA. Furthermore, the study highlights the importance of collaborative efforts at the national and global levels to curtail inappropriate antimicrobial use. Future research should focus on intervention-based studies to assess the efficacy of public health measures in reducing SMA prevalence and its contribution to AMR. A holistic, interdisciplinary approach integrating healthcare providers, policymakers, and the community remains essential in addressing this public health challenge.
